# Shikonin Inhibits Non-Small-Cell Lung Cancer H1299 Cell Growth through Survivin Signaling Pathway

**DOI:** 10.1155/2021/6435393

**Published:** 2021-11-06

**Authors:** Haini Wang, Junli Zuo

**Affiliations:** ^1^Respiratory Medicine ICU, Xi'an International Medical Center Hospital, Xi'an, Shaanxi 710000, China; ^2^Respiratory Medicine Ward, Xi'an International Medical Center Hospital, Xi'an, Shaanxi 710000, China

## Abstract

Overexpressed survivin is associated with worse survival of several types of human tumors. In this study, the antitumor activity of shikonin in non-small-cell lung cancer (NSCLC) by regulating survivin pathway was investigated. Results showed that shikonin inhibited the NSCLC H1299 cell proliferation in a dose-dependent manner. Moreover, shikonin fits well with survivin by molecular docking. Shikonin also inhibited the mRNA expression and protein level of survivin in H1299 cells. Shikonin arrested H1299 cell cycle at the G0/G1 phase by regulating CDK/cyclin family members. In addition, shikonin regulated the expression of X-linked inhibitor of apoptosis- (XIAP-) mediated caspases 3 and 9, thus leading to the damage of mitochondrial membrane potential and induction of H1299 cell apoptosis. Overall, shikonin inhibited H1299 cell growth by inducing apoptosis and blocking the cell cycle. The underlying mechanism involves targeting survivin, which subsequently regulates the protein expression of XIAP/caspase 3/9, CDK2/4, and cyclin E/D1. Thus, shikonin, a survivin inhibitor, is a promising therapeutic strategy in NSCLC treatment.

## 1. Introduction

Lung cancer is one of the deadliest cancers worldwide. More than 1.5 million people die from lung cancer each year. Lung adenocarcinoma, a common type of lung cancer, has a higher incidence rate than squamous cell carcinoma [1, 2]. Treating cancer with chemotherapy can reduce the risk of cancer-related deaths. Unfortunately, although new chemotherapy drugs enter the market every year, the adverse reactions and toxicity of these drugs have become a bottleneck in the treatment of clinical lung cancer [3]. Therefore, it is necessary to develop new treatment strategies to prevent the metastasis of lung adenocarcinoma.

Survivin is the smallest member of the inhibitor of apoptosis (IAP) protein family, which is regulated by the cell cycle and is expressed in the G2/M phase of the cell cycle [4]. Survivin inhibits the activation of caspases by binding to X-linked inhibitor of apoptosis (XIAP) protein, thereby inhibiting the apoptosis of cancer cells. Overexpression of survivin has carcinogenic potential because it can overcome the G2/M checkpoint and promote cell development through mitosis, which is conducive to the development of tumor clones [5]. It has also been reported that survivin can induce exit from G1 phase entry into S phase, and inhibition of survivin can arrest G0/G1 cell cycle in NSCLC cells [6, 7]. Therefore, survivin is a potential target of cancer.

Many studies have reported the antitumor activity of different Chinese medicines, revealing novel pathways for natural pharmacological compounds to prevent cancer. Various natural products used to treat different diseases, including cancer, are becoming increasingly important for drug discovery and development [8–11]. *Lithospermum erythrorhizon* is a common medicinal material in traditional Chinese medicine and has been used clinically for centuries in China. *Lithospermum erythrorhizon* has various pharmacological effects, including anti-inflammatory, antibacterial, and anticough effects. Researchers have isolated various active ingredients from *Lithospermum erythrorhizon*, including shikonin, acetylshikonin, and isobutylshikonin. Shikonin, one of the main active ingredients, has been reported to have a variety of pharmacological effects, including antitumor, antipyretic, analgesic, antifungal, antibacterial, and wound healing properties [12].

This study investigated the antitumor activity of shikonin in lung cancer cells H1299 and explored its antitumor molecular mechanism.

## 2. Materials and Methods

### 2.1. Chemicals and Reagents

**Figure 1:**
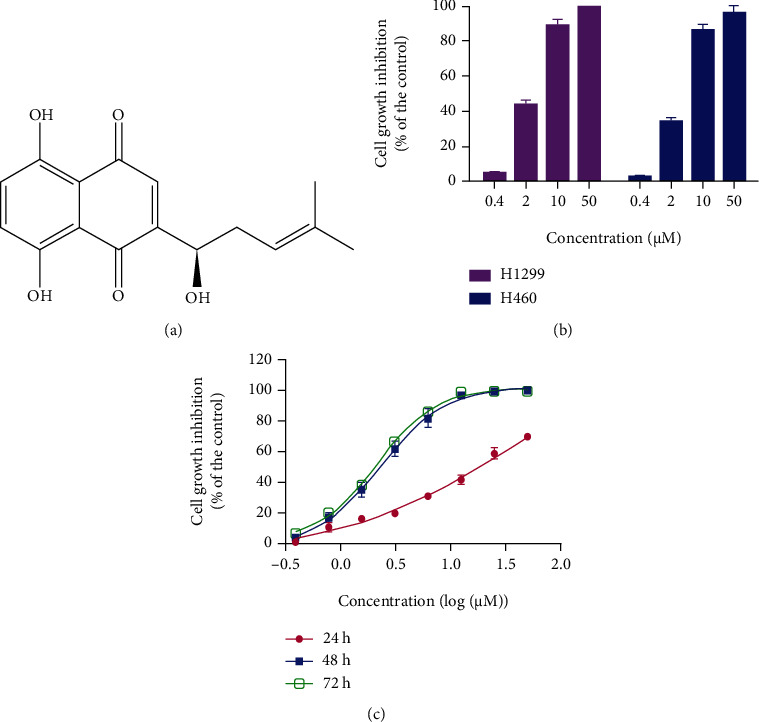
The effect of shikonin on NSCLC cell growth: (a) chemical structure of shikonin; (b) effect of shikonin on NSCLC cell (H1299 and H460) growth at 48 h; (c) effect of shikonin on H1299 cell growth at different time points. Cells treated with various concentrations of shikonin for 24, 48, and 72 h were measured by MTT assay. Five replicates for each treatment were done, and the data are presented as the mean ± standard error of the mean from three repeated experiments.

Shikonin (Figure 1(a)) was purchased from Weikeqi Biotech (Chengdu, China). H1299 and H460 cells were obtained from the Shanghai Institute of Cell Biology at the Chinese Academy of Sciences (Shanghai, China). RPMI 1640 medium, trypsin, and dimethylsulfoxide (DMSO) were purchased from Sigma-Aldrich (St. Louis, MO, USA). CCK8 was from Zeta Life (Adams Drive, CA, USA). The RNAfast200 kit was purchased from Fastagen (Shanghai, China), and Lipofectamine 2000 reagent and JC-1 were purchased from Invitrogen (Carlsbad, CA, USA). PrimeScript RT Master Mix Perfect Real Time Kit, SYBR® Premix Ex Taq™ II, and a Thermal Cycler Dice Real Time System were purchased from TaKaRa (DRR036A) Biotechnology (Dalian, China). Annexin V-FITC apoptosis detection kit and Hoechst 33258 staining kit were purchased from Beyotime Institute of Biotechnology (Shanghai, China). Kinase-Glo Plus luminescence kinase assay kit was from Promega (Madison, WI, USA). PVDF membrane was from Millipore (Merck, Billerica, MA, USA). RNase and propidium iodide (PI) were from Sigma-Aldrich (St. Louis, MI, USA). Survivin, XIAP, cleaved caspase-3, caspase-3, cleaved caspase-9, caspase-9, CDK2, CDK4, cyclin E, and cyclin D1 antibodies were purchased from Cell Signaling Technology, Inc. (Danvers, MA, USA). GAPDH monoclonal antibody was purchased from Santa Cruz Biotechnology (Shanghai, China). HRP-conjugated goat anti-rabbit IgG, BCA protein assay reagent kit, and SuperSignal® West Pico were purchased from Pierce Biotechnology (Waltham, MA, USA). Protease inhibitor cocktail and phosphatase inhibitor cocktail were purchased from Roche (Basel, Switzerland).

### 2.2. Cell Culture

Human lung cancer cell lines H1299 and H460 were cultured in RPMI1640 medium containing 10% fetal bovine serum (FBS), 100 U/mL penicillin, 100 U/mL streptomycin (Invitrogen, Scotland, UK), and 2 mM L-glutamine at 37°C in a 5% CO_2_ incubator with saturated humidity.

### 2.3. Cell Viability Assay

CCK8 was used to detect the cell viability of shikonin against H1299 and H460 cells. Cells in the logarithmic growth phase were collected and seeded in a 96-well plate (5 × 10^4^ cells/well). After treatment with the indicated concentrations of shikonin and DMSO diluted in medium (volume ratio, 1 : 40) as the control group, CCK8 reagent was added, and the cells were incubated at 37°C for 1.5 h. The absorbance was measured at a wavelength of 450 nm to calculate the cell viability.

### 2.4. Flow Cytometry of the Cell Cycle

After treating H1299 cells with different concentrations of shikonin for 48 h, the cells were collected, washed with PBS, and fixed in ice-cold 70% ethanol overnight at 4°C. Cells treated with DMSO diluted in medium (volume ratio, 1 : 40) were considered as the control group. The cells were then washed with PBS and stained with RNase and PI for 30 min out of light. The cell cycle was analyzed using a BD FACSCalibur flow cytometry (Becton-Dickinson, Franklin Lakes, NJ, USA).

### 2.5. Hoechst Staining Assay

After treating H1299 cells with different concentrations of shikonin for 48 h, the cells were incubated with Hoechst 33258 at 37°C for 10 min according to the instructions. Then, the cells were examined using a fluorescence microscope.

### 2.6. Flow Cytometry of Cell Apoptosis

After treating H1299 cells with different concentrations of shikonin for 48 h, the cells were collected, washed with PBS, and resuspended. Cells treated with DMSO diluted in medium (volume ratio, 1 : 40) were considered as the control group. According to manufacturer's instructions in the apoptosis kit, the cells were double-stained with PI and Annexin V-FITC, treated at 4°C in the dark for 20 min, and detected by flow cytometry.

### 2.7. Measurement of Mitochondrial Transmembrane Potential (*Δψ*m)

After treating H1299 cells with different concentrations of shikonin for 48 h, the cells were collected, washed, and resuspended in RPMI 1640 medium. Cells were stained with 10 *μ*M JC-1 at 37°C for 30 min under dark conditions and washed again with RPMI 1640 medium. The fluorescence intensity was measured using a flow cytometer.

### 2.8. PCR

Total RNA was extracted using the RNAfast 200 kit, following the manufacturer's protocol. The RNA was converted into cDNA using the PrimeScript RT Master Mix. RT-PCR was performed using SYBR® Premix Ex Taq™ II as a fluorescence probe. The relative amount of survivin mRNA was normalized to that of GAPDH mRNA. The primer sequences were as follows: GAPDH forward primer: 5′-AAGGCTGTGGGCAAGGTCATC-3′; GAPDH reverse primer: 5′-GCGTCAAAGGTGGAGGAGTGG-3′; survivin forward primer: 5′-CCAGATGACGACCCCATAGAG-3′; survivin reverse primer: 5′-TTGTTGGTTTCCTTTGCAATTTT-3′.

### 2.9. Western Blotting

H1299 cells were treated with shikonin at different concentrations, after 48 h, cells were harvested. Cells treated with DMSO diluted in medium (volume ratio, 1 : 40) were considered as the control group. Cells were lysed with RIPA lysis buffer containing protease and phosphatase inhibitors for 30 min. BCA detects protein content. The same amount of protein was separated by 10-15% SDS-PAGE and transferred to a PVDF membrane. The membranes were blocked with Tris-buffered saline containing 0.05% Tween-20 (TBST) and 5% low-fat powdered milk for 1 h. The blot was then incubated with the primary antibody overnight at 4°C. After washing with TBST for 10 min three times, the blots were incubated with a secondary antibody for 1 h at 37°C. The blot was washed three times with TBST before exposure to the SuperSignal West Dura Extended Duration substrate. Band intensity was quantified by densitometric analysis using a densitometer.

### 2.10. Molecular Docking

To investigate the interactions between the receptor and ligand, molecular docking studies were conducted using the SurflexDockMode of SYBYL-X 2.0 program package (New Tripos International, St. Louis, USA). The docking model of survivin protein (PBD ID: 1XOX) used in the study was retrieved from the protein databank.

### 2.11. Statistical Analysis

All values were expressed as means ± standard error of means (SEM). Statistics was determined with ANOVA. Results were considered statistically significant if the *P* value was <0.05.

## 3. Results

### 3.1. Shikonin Inhibits NSCLC Cell Proliferation

CCK8 assay was used to detect the inhibitory effect of shikonin on the proliferation of NSCLC cells (H1299 and H460 cells). Results showed that shikonin inhibited both H1299 and H460 cell proliferation and that H1299 cells were slightly more sensitive to shikonin than H460 cells (Figure 1(b)). Therefore, H1299 cells were used for further studies. In addition, shikonin inhibited the growth of H1299 cells in a dose- and time-dependent manner (Figure 1(c)). The IC_50_ values of shikonin treatment for 24, 48, and 72 h were >50 *μ*M, 2.32 *μ*M, and 2.15 *μ*M, respectively.

### 3.2. Shikonin Arrests H1299 Cell Cycle at the G0/G1 Phase

**Figure 2:**
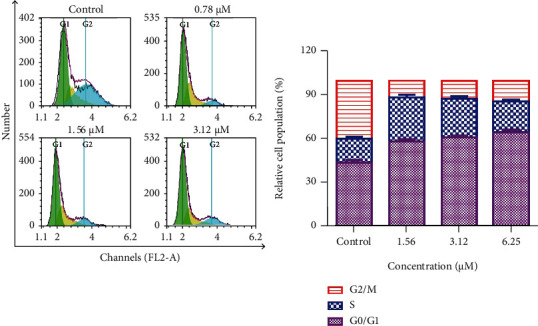
Effect of shikonin on cell cycle distribution. H1299 cells were treated with shikonin (0, 0.78, 1.56, and 3.12 *μ*M) for 48 h followed by staining with propidium iodide for flow cytometric analysis. Data are presented as the mean ± standard error of the mean (*n* = 3).

To determine the effect of shikonin on the H1299 cell cycle, flow cytometry was performed. Results showed that shikonin treatment arrested H1299 cells in the G0/G1 phase (Figure 2). With the increasing concentration of shikonin from 0.78 *μ*M, 1.56 *μ*M to 3.12 *μ*M, the H1299 cell population in the G0/G1 phase increased to 58.25%, 60.93%, and 64.50% compared with the control group (43.83%).

### 3.3. Shikonin Induces Apoptosis in H1299 Cells

**Figure 3:**
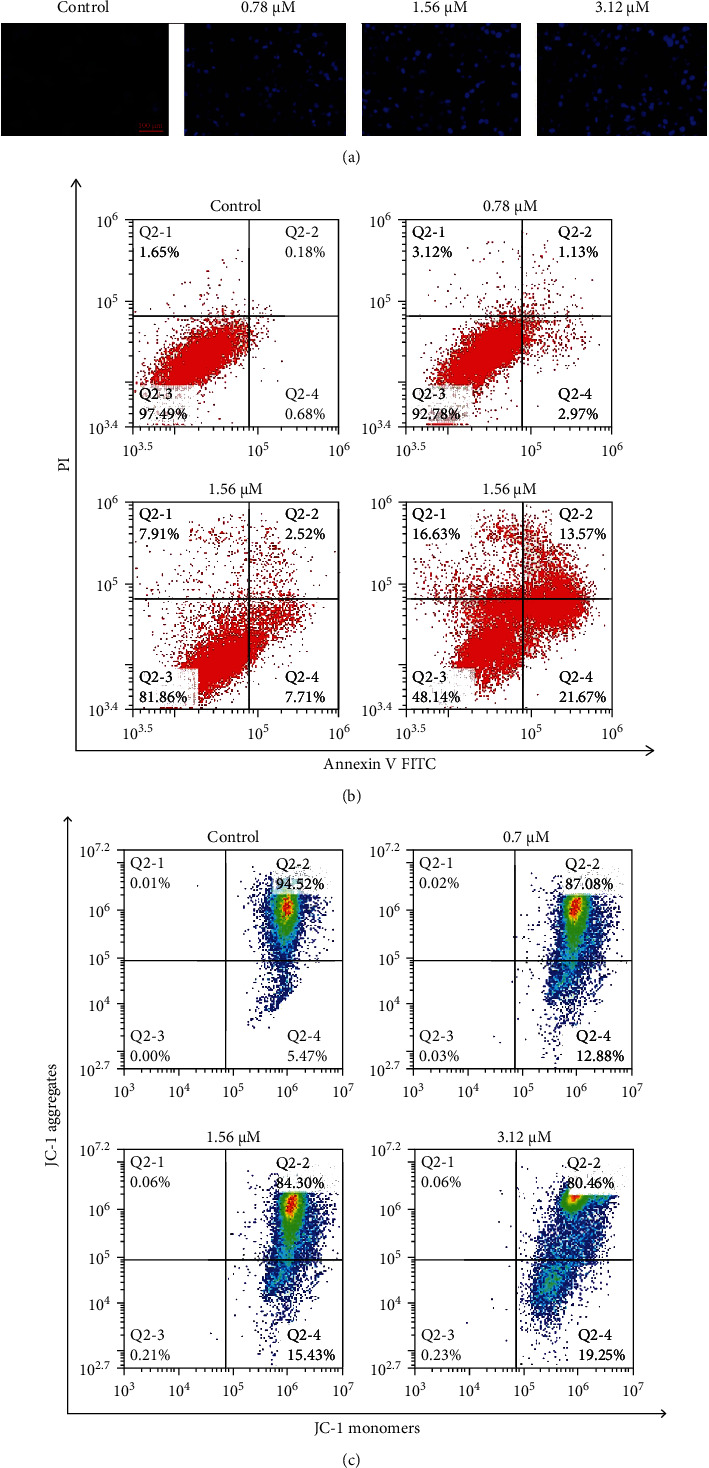
Effect of shikonin on cell apoptosis. (a) Shikonin-induced apoptosis in H1299 cells was characterized by nuclear condensation or nuclear fragmentation after Hoechst staining. (b) Flow cytometric analysis of shikonin-induced apoptosis in H1299 cells. The flow cytometry profile presents Annexin V FITC (*x*-axis) and PI staining (*y*-axis). The values represent the percentage of cells in each of the four quadrants (lower left quadrant, viable cells; upper left quadrant, necrotic or dead cells; lower right quadrant, early stage apoptotic cells; and upper right quadrant, late stage apoptotic cells). (c) The mitochondrial membrane potential (*Δψ*m) was assessed using flow cytometry following the treatment of H1299 cells with shikonin (0.78, 1.56, or 3.12 *μ*M) for 48 h.

To determine the effect of shikonin on cell apoptosis, H1299 cells were stained with Hoechst 33258 dye. As shown in Figure 3(a), Hoechst staining of H1299 cells showed that shikonin led to chromatin condensation, indicating the formation of nuclear apoptotic bodies. Microscopic observation of Figure 3(a) shows Hoechst staining of typical apoptotic cell nuclei. Chromatin also aggregated on the nuclear membrane, as indicated by the bright fluorescence at the periphery. Flow cytometry was used to determine the rate of apoptosis. After shikonin treatment, an increase in the number of Annexin V-positive cells was observed (Figure 3(b)). The apoptotic percentage of H1299 cells treated with shikonin increased to 4.10%, 10.23%, and 35.24% compared with the control group (0.86%).

Mitochondrial transmembrane potential (*Δψ*m) was determined by flow cytometry. JC-1 staining showed that the *Δψ*m of H1299 cells treated with shikonin was significantly reduced. The inhibition of JC-1 mitochondrial accumulation and the reduction of JC-1 aggregation formation indicated alterations in mitochondrial function (Figure 3(c)).

### 3.4. Effects of Shikonin on Survival

**Figure 4:**
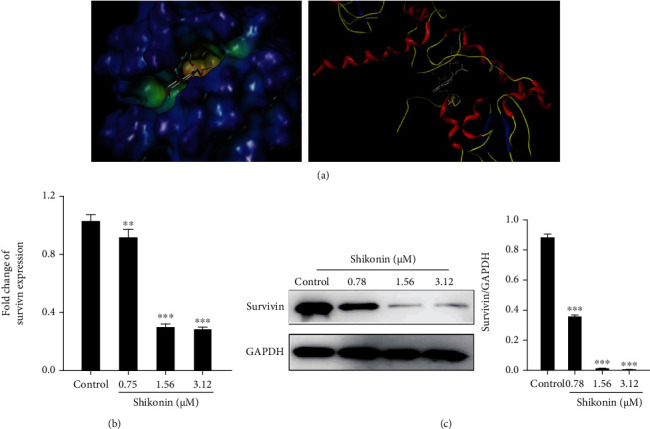
Effect of shikonin on survivin. (a) Docked molecule (shikonin) in the crystal structure of survivin (PDB ID: 1XOX). Hydrogen bonds were depicted as dashed yellow lines. (b) The mRNA level of survivin in H1299 cells treated with shikonin (0, 0.78, 1.56, and 3.12 *μ*M) for 48 h. (c) The protein level of survivin in H1299 cells treated with shikonin (0, 0.78, 1.56, and 3.12 *μ*M) for 48 h was examined by western blot assay. Data are presented as the mean ± standard error of the mean (*n* = 3). ^∗∗^*P* < 0.01 and ^∗∗∗^*P* < 0.001*vs.* control.

As survivin plays an important role in tumor growth, the effect of shikonin on survivin was evaluated. Molecular docking studies were used to investigate the ligand-protein interactions between survivin and shikonin.  Figure 4(a) shows that the sphere space field model revealed that shikonin has a good fit within the active cavity of survivin. In addition, shikonin occupied the active pocket of survivin with three hydrogen bonds by binding to LYS15, LYS91, and GLN92 with distance of 2.74 Å, 2.09 Å, and 1.97 Å. In addition, shikonin inhibited the expression of survivin mRNA (Figure 4(b)) and decreased the protein levels of survivin in H1299 cells (Figure 4(c)). These data indicated that shikonin could target survivin.

### 3.5. Shikonin Regulates the Survivin-Related Signaling Pathway

**Figure 5:**
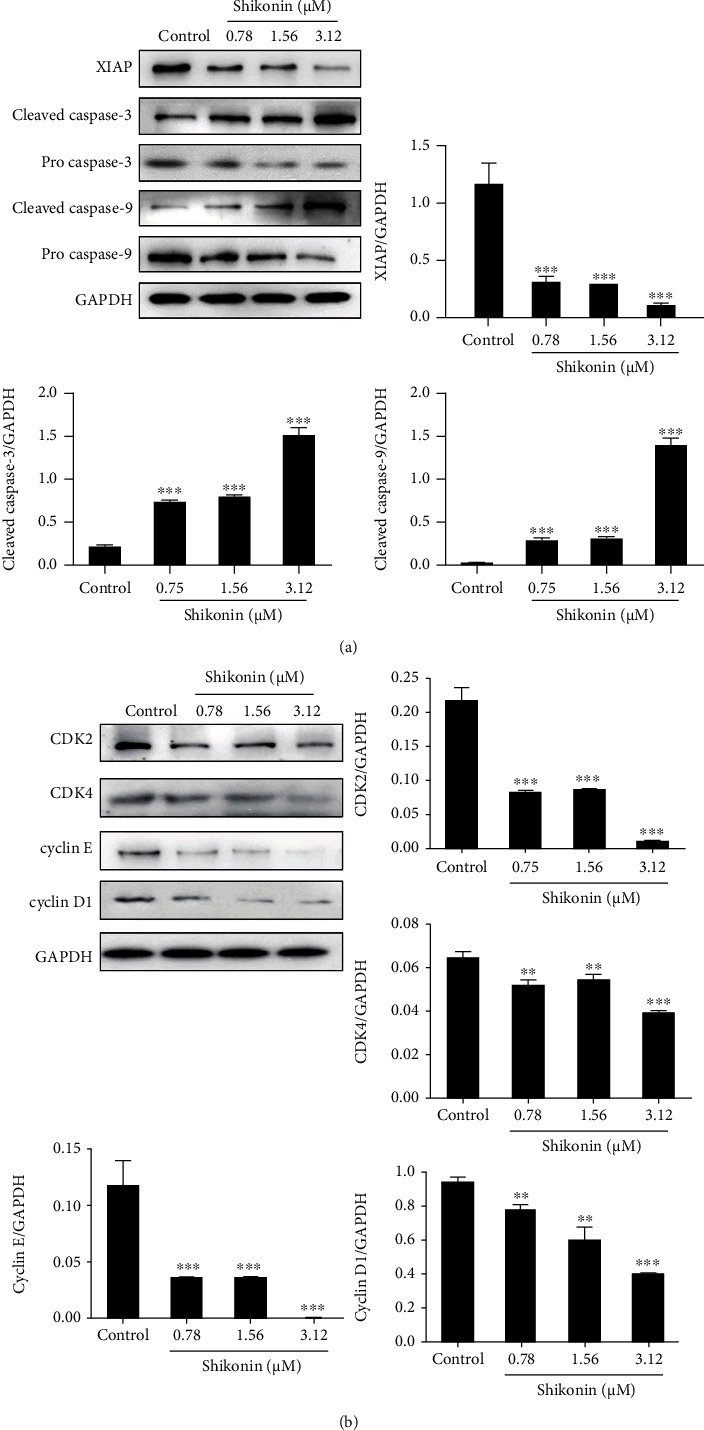
Effect of shikonin on molecules of cell apoptosis and cycle-related signaling. (a) Protein levels of XIAP and cleaved caspases 3 and 9 in H1299 cells treated with shikonin (0, 0.78, 1.56, and 3.12 *μ*M) for 48 h were examined by western blot assay. (b) The protein levels of CDK2, CDK4, cyclin E, and cyclin D1 in H1299 cells treated with shikonin (0, 0.78, 1.56, and 3.12 *μ*M) for 48 h were examined by western blot assay. Data are presented as the mean ± standard error of the mean (*n* = 3). ^∗^*P* < 0.05, ^∗∗^*P* < 0.01, and ^∗∗∗^*P* < 0.001*vs.* control.

Targeting survivin could regulate the cell cycle and apoptosis. Therefore, we detected the major molecules in the major downstream signaling pathway. Shikonin treatment significantly downregulated the protein levels of XIAP and increased the protein levels of cleaved caspases 3 and 9 (Figure 5(a)). In addition, shikonin treatment downregulated the protein levels of CDK2, CDK4, cyclin E, and cyclin D1 (Figure 5(b)).

## 4. Discussion

Lung cancer is the most common cause of cancer death worldwide, and approximately 85% of lung cancer cases are characterized as NSCLC [13, 14]. Shikonin is a natural naphthoquinone compound isolated from the traditional Chinese medicine *Lithospermum erythrorhizon*, and it has been reported to treat various diseases, including viral infections, inflammation, and cancer [15]. However, whether shikonin exerts its anticancer effects through survivin has not been reported previously. In this study, we demonstrated that shikonin inhibited H1299 cell proliferation by blocking the cell cycle and inducing cell apoptosis, which were regulated by targeting survivin.

The results showed that shikonin inhibited NSCLC (H1299 and H460) cell growth, and the sensitivity of H1299 cells to shikonin was higher than that of H460 cells. In addition, the inhibitory effect of shikonin on H1299 cells was dose-dependent. Because the effect of shikonin was almost similar at 48 h and 72 h, we selected a shorter time point for further analysis.

Survivin is the smallest member of the IAP family of proteins and plays an important role in cancer development [16, 17]. Survivin has been reported to be overexpressed and associated with poor survival in several types of human tumors [18, 19]. Inhibition of survivin can induce apoptosis and inhibit the proliferation and invasion of cancer cells [20]. Thus, survivin is considered a good target for cancer treatment. In this study, shikonin was found to fit well with survivin by molecular docking. Our molecular docking results revealed that shikonin has the potential to directly target survivin, while further direct experiments are required to assess the relationship between shikonin and survivin. In addition, shikonin inhibited the expression of survivin mRNA and the protein level of survivin in H1299 cells. These data indicate that shikonin may target survivin.

Studies have reported that survivin overexpression could promote cell cycle progression, leading to uncontrolled proliferation of cancer cells [21]. Our results showed that shikonin treatment arrested the cell cycle at the G0/G1 phase in H1299 cells, and a previous study has also reported that shikonin induces G0/G1 phase arrest in gallbladder cancer cells [22]. As survivin inhibition can induce G0/G1 cell cycle arrest in NSCLC cells [6, 7], we speculated that shikonin arrested the cell cycle through survivin. Cyclins (cyclin D1 and cyclin E) and CDKS (CDK4, CDK6, and CDK2) are the key mediators in the G1 to S phase transition [23], which can be regulated by survivin [24, 25]. Our results confirmed that shikonin treatment could downregulate the protein levels of CDK2, CDK4, cyclin E, and cyclin D1 in H1299 cells, inducing G0/G1 cell cycle arrest.

As a member of the IAP family, survivin plays a critical role in promoting cell proliferation and blocking cell apoptosis [26]. Our study evaluated the effect of shikonin on the induction of apoptosis in H1299 cells. Results showed that cells treated with shikonin exhibited chromatin condensation, and shikonin induced apoptosis in H1299 cells in a dose-dependent manner. Furthermore, shikonin treatment reduced the *Δψ*m in H1299 cells. The mechanism of the inhibitory effect of survivin on apoptosis involves the direct inhibition of caspases. Survivin could enhance the XIAP-mediated caspase 3 and 9 inhibition, which is the downstream terminal effector of the apoptotic pathway [18, 20, 27]. Our results confirmed that shikonin treatment significantly upregulated the protein levels of XIAP and downregulated the protein levels of cleaved caspases 3 and 9.

## 5. Conclusion

This study demonstrated that shikonin inhibited H1299 cell growth, induced cell apoptosis, and blocked the cell cycle. The underlying mechanism involves targeting survivin, which subsequently regulates the protein expression of apoptosis-related and CDK/cyclin family members. Thus, shikonin, a survivin inhibitor, is a promising therapeutic strategy for NSCLC treatment.

## Data Availability

The data used to support the findings of this study are included within the article.
